# Landscape and Treatment Options of Shapeshifting Small Cell Lung Cancer

**DOI:** 10.3390/jcm13113120

**Published:** 2024-05-26

**Authors:** Yijun Gu, Claudia A. Benavente

**Affiliations:** 1 Department of Pharmaceutical Sciences, University of California, Irvine, CA 92697, USA; yijung3@uci.edu; 2 Department of Developmental and Cell Biology, University of California, Irvine, CA 92697, USA; 3 Chao Family Comprehensive Cancer Center, University of California, Irvine, CA 92697, USA

**Keywords:** small cell lung cancer, tumor plasticity, immunotherapy, targeted therapy

## Abstract

Small cell lung cancer (SCLC) is a deadly neuroendocrine malignancy, notorious for its rapid tumor growth, early metastasis, and relatively “cold” immune environment. Only standard chemotherapies and a few immune checkpoint inhibitors have been approved for SCLC treatment, revealing an urgent need for novel therapeutic approaches. Moreover, SCLC has been recently recognized as a malignancy with high intratumoral and intertumoral heterogeneity, which explains the modest response rate in some patients and the early relapse. Molecular subtypes defined by the expression of lineage-specific transcription factors (ASCL1, NEUROD1, POU2F3, and, in some studies, YAP1) or immune-related genes display different degrees of neuroendocrine differentiation, immune cell infiltration, and response to treatment. Despite the complexity of this malignancy, a few biomarkers and targets have been identified and many promising drugs are currently undergoing clinical trials. In this review, we integrate the current progress on the genomic landscape of this shapeshifting malignancy, the characteristics and treatment vulnerabilities of each subtype, and promising drugs in clinical phases.

## 1. Introduction

Lung cancer is the most common cancer worldwide, with a high incidence (11.6%) and mortality rate (18.4%) in both men and women [1]. Small cell lung cancer (SCLC) constitutes 15% of all lung cancer cases and is known for its aggressive and highly metastatic nature [2]. SCLC typically originates in the bronchi and is most frequently associated with smoking. The incidence rate of SCLC was 9.5 per 10,000 population in 1975 and peaked at 15.3 in 1988. Since the initiation of tobacco control programs around the world, the incidence of SCLC declined to 6.5 in 2019 [3]. Unfortunately, SCLC often goes undiagnosed until it reaches an advanced state due to its rapid tumor growth, lack of symptoms, and symptoms being mistaken for those caused by smoking and air pollution. The percentage of advanced stage IV at diagnosis has increased from 58.6% in 1988 to 70.8% in 2010 [3]. As a result, the 5-year survival rate for SCLC patients is less than 6% in approximately 70% of cases [2].

SCLC is a high-grade neuroendocrine tumor that exhibits abnormal neurotransmitter or hormone signaling [4]. This is thought to be linked to genetic variations, such as the inactivation of *TP53* and *RB1*, as well as disruptions in signaling pathways. The neuroendocrine nature of SCLC has opened up new avenues for targeted therapy and immunotherapy. Several clinical studies are currently ongoing with some promising results. 

In addition to developing new drugs for known targets, substantial efforts have been invested in understanding and categorizing different SCLC subtypes. Traditionally treated as a single disease, recent evidence suggests significant heterogeneity in the degree of neuroendocrine differentiation and the regulation of neuronal lineage-specific transcription factors within SCLC. This has led to the identification of several SCLC subtypes, including SCLC-A (ASCL1-positive), SCLC-N (NEUROD1-positive), SCLC-P (POU2F3-positive), and SCLC-Y (YAP1-positive) or SCLC-inflamed [5,6]. 

This review aims to provide an overview of SCLC’s genomic variations, differences among its subtypes, and a summary of current and emerging therapeutic and diagnostic strategies. The goal is to assess the most promising approaches to improving outcomes for SCLC patients.

## 2. Unveiling the Genomic Landscape of SCLC

In 1988, a pivotal discovery was made by Harbour and colleagues: they found that the *RB1* gene was exclusively inactivated in SCLC, setting it apart from non-SCLC cell lines and normal human lungs [7]. Shortly thereafter, in 1989, Takahashi and colleagues identified abnormal *TP53* mutations in lung cancer cells [8]. This paved the way for the confirmation that SCLC is characterized by the nearly universal inactivation of both *TP53* and *RB1*. This inactivation is believed to be the catalyst for subsequent genomic and epigenetic alterations that bestow SCLC with its neuroendocrine characteristics [9].

Fast forwarding to 2010, Pleasance and colleagues achieved a remarkable milestone by conducting the first whole genome sequencing of an SCLC cell line, NCI-H209. This cell line was derived from a bone marrow metastasis of a 55-year-old male with SCLC before chemotherapy, and the sequencing revealed that tobacco smoking significantly amplifies the mutational burden in SCLC. Additionally, it unveiled various tumor signatures, including *APOBEC*, a cytidine deaminase involved in RNA editing [10,11].

Following Pleasance’s groundbreaking work, advanced sequencing tools have empowered researchers to create more comprehensive genomic profiles of SCLC [12]. A deeper analysis of these genomic alterations revealed that many of the mutated genes in SCLC can be grouped into four main categories: regulators of cell cycle and death, epigenetic regulators, members of the Notch signaling pathway, and regulators of cytoskeleton dynamics and cell adhesion [12]. 

As we continue to explore these genomic and epigenomic changes, significant efforts have been invested in identifying potential biomarkers and developing targeted therapies against them. 

### 2.1. Loss of the RB and TP53 Families in SCLC

In SCLC, the loss of *RB1* and *TP53* is nearly universal, but it does not stop there. The inactivation of their homologs, like *RBL1* (3–4%), *RBL2* (5–7%), and *TP73* (13%), has also been reported in SCLC [5]. *RBL1* and *RBL2*, also known as *p107* and *p130*, respectively, share crucial functions with RB1, regulating E2F transcription factors that control the cell cycle. It has been suggested that RBL1 and RBL2 may compensate for the functional loss of other RB family members, contributing to the latent development of tumors in SCLC. This idea gained support in a study where *Rb1*/*Tp53*-mutant genetically engineered mouse models (GEMMs) with the homozygous or heterozygous deletion of *Rbl2* showed significantly higher tumor incidence and shorter tumor latency compared to those with wild-type *Rbl2* [13]. Similarly, *TP73*, along with *p63*, comprises the p53 family of transcription factors, which controls cell cycle arrest and apoptosis by inducing the expression of related genes [14]. 

Although the mutational pattern of TP53 aligns with what is expected from tobacco exposure, there is no direct evidence linking tobacco exposure to *RB1* loss in SCLC [15,16]. Interestingly, even though *RB1* is among the significantly mutated genes in SCLC, approximately 10% of patients do not exhibit *RB1* mutations [12]. The RB1 protein undergoes initial mono-phosphorylation mediated by the CDK4/6-Cyclin D complex during the G1 phase, leading to the release of the E2F transcription factors. Studies have revealed that SCLC tumors with functional RB1 protein are more responsive to CDK4/6 inhibitors like palbociclib and abemaciclib, and this sensitivity is dependent on the presence of RB1 [17]. 

### 2.2. Rare Kinase Alterations in SCLC

A genome-wide analysis of the kinome reveals that kinases have diverse biological functions and play crucial roles in oncogenesis, with kinase inhibitors accounting for a significant portion of small molecule inhibitors used in cancer treatment [18]. Unfortunately, in comparison to non-small cell lung cancer (NSCLC), SCLC exhibits fewer kinase mutations. Commonly mutated kinases in NSCLC, such as epidermal growth factor receptor (EGFR) and MAPK pathway kinases (KRAS, RAF, MEK, and ERK), are seldom mutated in SCLC [12]. However, SCLC patients who do have kinase mutations might benefit from genetic sequencing and targeted kinase inhibitors.

Two well-known receptor tyrosine kinases with abnormal expression in SCLC are c-Kit (or CD117) and fibroblast growth factor receptor 1 (FGFR1). C-Kit is a proto-oncogene that encodes a tyrosine kinase receptor and is believed to create an autocrine loop that drives cell proliferation with its ligand stem cell factor (SCF) [19]. Positive c-Kit expression has been detected in 37% of SCLC patient samples and is associated with reduced survival [20]. Activating mutations of c-Kit have also been identified, with S476I and P551A, mutations occurring in 3.3% and 5% of cases, respectively [12,21]. Imatinib mesylate (Gleevec), a small molecule inhibitor for several tyrosine kinase receptors, including c-Kit, has shown efficacy in gastrointestinal cancer and acute lymphoblastic leukemia linked to c-Kit S476I and P551A mutations. However, in a phase II clinical trial, imatinib mesylate failed to demonstrate therapeutic efficacy in SCLC patients [22], possibly due to the low incidence of these mutations. Nevertheless, alternative approaches to target c-Kit are being explored [23]. 

In contrast, the ectopic expression of *Fgfr1* in precancerous neuroendocrine cells (preSCs) has been found to increase in vitro cell growth and tumor formation in immune-compromised mice, along with enhanced proliferation-related gene expression changes [24]. *FGFR1* amplification has been reported in a subset of human SCLC tumor samples (6–30%). The study in [12] and subsequent research revealed that the constitutive activation of FGFR1 inhibits SCLC initiated from calcitonin gene-related peptide (CGRP)-positive neuroendocrine cells but promotes SCLC initiated from K14-positive tracheobronchial-basal epithelial cells. This suggests that SCLC may originate from a more diverse set of cell lineages than previously assumed, and the role of FGFR1 is context-dependent [25]. 

Aurora kinases A and B (AURKA and AURKB) are key serine/threonine kinases that regulate mitosis and coordinate the G2-M transition [26]. The overexpression of Aurora kinases has been reported in various cancers, including lung cancer [27]. Small molecule inhibitors of AURKA and AURKB have shown antitumor activity by inducing polyploidy in SCLC cell lines with *MYC* amplification and *RB1* inactivation [28]. The effectiveness of these inhibitors correlated with the levels of *MYC* amplification and expression, making Aurora kinases promising therapeutic targets in SCLC with high *MYC* expression. Additionally, other kinases involved in cell cycle and DNA repair, such as ATR and WEE1, have emerged as promising targets, with several inhibitors in clinical trials [29,30]. 

### 2.3. MYC Amplification in SCLC

The amplification of *MYC* family genes, including *MYC*, *MYCL*, and *MYCN*, is a common oncogenic event in SCLC [31]. Approximately 20% of SCLC tumors and 30–50% of SCLC cell lines exhibit MYC amplification [31]. This genetic alteration is associated with a grim prognosis in SCLC patients, reducing their survival from 26 weeks to just 4 weeks [32]. 

SCLC cell lines with frequent *MYC* amplification tend to have a faster doubling time, indicating more aggressive growth [31]. The expression of *Myc* family members, especially *Mycl*, can restore the tumor growth capacity in non-tumorigenic, preneoplastic SCLC cells within weeks [33]. Conversely, inhibiting *MYC* amplification in SCLC cell lines hampers tumor cell growth [34]. 

In vivo studies utilizing GEMMs also shed light on the potential tumorigenic role of *Myc* in SCLC. One study involved creating experimental *Rb1^fl/fl^ p53^fl/fl^ Myc^LSL/LSL^* (RMP) mice by crossing *Rb1^fl/fl^ p53^fl/fl^* (RP) mice with mice carrying Lox-Stop-Lox (LSL)-MycT58A-IRES-Luciferase and having a Cre-MycT58A recombinase [31]. RPM mice were infected intratracheally with adenoviruses containing Cre driven by the CGRP promoter, a marker expressed only in the predominant cell of origin of SCLC in the RP model [35]. RPM mice experienced significantly higher mortality compared to *Rb1^fl/fl^ Trp53^fl/fl^ Pten^fl/fl^* (RPP) mice, with a median survival of 60 versus 164 days, respectively [36,37]. Heterozygous RPM mice (*Rb1^fl/fl^ Trp53^fl/fl^ Myc^LSL/+^*) had a slightly longer median survival of 81 days than RPM mice. Although the precise mechanism remains unclear, both in vitro and in vivo studies suggest that *MYC* amplification accelerates tumor growth, thereby shortening the survival of SCLC patients. This indicates that *MYC* could be a potential therapeutic target in the *MYC*-amplified SCLC subset. 

Furthermore, increasing evidence suggests that MYC may play a role in driving the evolution of SCLC subtypes by regulating neuroendocrine and metabolic processes [31,38,39]. This aspect will be explored in more detail in a later section. 

### 2.4. Notch Signaling Pathway in SCLC

The Notch signaling pathway plays a critical role in cell–cell communication, regulating transcription, and cell differentiation. In normal cells, the transcription factor achaete-scute homolog 1 (ASCL1) induces neuroendocrine differentiation, leading to the expression of neuroendocrine markers and activating the transcription of *DLL3* and other genes in the Notch pathway. However, in lung cancers, the role of Notch pathway genes can vary, with their main function being that of tumor suppressors in SCLC [40]. 

A comprehensive study of SCLC cases involving genome sequencing revealed mutations in Notch family genes in approximately 25% of the cases examined [2], and these genes are generally suppressed in the majority of neuroendocrine SCLC cases [40]. Yet, recent research suggests that Notch signaling may also have a pro-tumorigenic role in SCLC. The non-neuroendocrine subtype of SCLC, which has an active Notch pathway, tends to be more resistant to chemotherapy and can support the growth of neighboring neuroendocrine SCLC cells [41]. 

There are three known mechanisms for inactivating the Notch pathway in SCLC: (1) the mutational inactivation of Notch pathway genes; (2) the inhibition of Notch receptors by canonical Notch ligand Delta-like ligand 3 (DLL3) or Delta-like non-canonical Notch ligand 1 (DLK1); and (3) the degradation of Notch receptors guided by DLL3 in the endosomes [42].

As a result, Notch pathway proteins, especially DLL3, are considered potential therapeutic targets. DLL3 is exclusively overexpressed in SCLC. However, the development of the experimental drug Rovalpituzumab tesirine (Rova-T), a DLL3-targeted antibody–drug conjugate, was halted after the phase III trial due to its lack of significant benefit and toxicity [43]. This could be partly attributed to the toxicity of the anti-cancer payload pyrrolobenzodiazepine (PBD). Presently, alternative approaches using DLL3 for T cell-redirecting therapies are in clinical trials [44], and the experience gained from these therapies will help determine whether DLL3 or other Notch pathway inhibitors are promising targets in SCLC, considering the pro-tumorigenic role of the Notch pathway in the non-neuroendocrine subgroup. 

### 2.5. Epigenetic Alterations in SCLC

SCLC presents a unique epigenetic regulation pattern for DNA methylation, histone methylation, and histone acetylation when compared to other lung cancers. This distinct pattern offers an opportunity for the development of SCLC-targeted epigenetic drugs. One of the most crucial and extensively studied oncogenes related to histone methylation is the Enhancer of Zeste Homolog 2 (EZH2). EZH2 is the enzymatic catalytic subunit of Polycomb Repressive Complex 2 (PRC2), responsible for tri-methylating H3K27 and silencing gene expression. In SCLC, due to the universal loss of RB1, EZH2 is expressed at higher levels compared to NSCLC and normal lung epithelial cells [45]. EZH2 has been found to epigenetically silence the TGF-ß type II receptor (TßRII) and suppress the TGF-ß-Smad-ASCL1 pathway, resulting in elevated ASCL1 expression and promoting SCLC progression [45]. Another study demonstrated that EZH2 also drives acquired chemoresistance in relapsed SCLC through the EZH2-SLFN11 axis [46]. These promising preclinical findings have led to phase I/II clinical trials of EZH2 inhibitor DS-3201b in combination with irinotecan and a phase I trial of another EZH2 inhibitor, PF-06821497, in patients with recurrent SCLC [47,48]. 

Another potential epigenetic target related to histone methylation is Lysine-specific demethylase 1A (LSD1). LSD1 inhibitors have shown SCLC-specific activity by reactivating the Notch pathway and reducing the expression of ASCL1 and neuroendocrine lineage genes [49]. Currently, the LSD inhibitor CC-90011 is undergoing two clinical trials, one in combination with standard chemotherapies and another with Nivolumab for SCLC [50,51]. 

CREB-binding protein (*CREBBP*) and E1A-associated p300 (*EP300*) are lysine acetyltransferases (KATs) and are two major inactivated genes in SCLC, with a mutation frequency of 15% and 13%, respectively [12]. These two proteins mediate the acetylation of H3K27, which leads to the transcription of regulated downstream genes. The majority of these hotspot inactivation mutations occur in KAT domains, resulting in decreased chromatin accessibility. These mutations in *CREBBP* and *EP300* have a mutually exclusive pattern, indicating they may share similar functions in SCLC development. The functional loss of *CREBBP* and *EP300* has been linked to various neuroendocrine cancers by activating oncogenes and suppressing tumor suppressor genes through epigenetic modifications [5]. One study demonstrated that *Crebbp* loss resulted in the reduced expression of tight junction and cell adhesion genes, including *Cdh1*, in SCLC mouse models [52]. The treatment of lysine deacetylase (KDAC) inhibitor was able to restore histone acetylation and the expression of CDH1 [52]. Several KDAC inhibitors are currently in phase I trials as a monotherapy or in combination therapies [53,54,55]. Given the diverse role and reversible alterations of epigenetics in cancer, targeting epigenome in SCLC is a very promising treatment strategy. 

### 2.6. Alterations of Cytoskeletal and Cell Adhesion Genes in SCLC

While SCLC is notorious for its frequent metastasis, the precise mechanisms by which genetic and transcriptional alterations affect metastasis are not fully understood. The whole-genome sequencing of clinical SCLC samples has revealed somatic mutations in genes such as *ALMS1*, *ASPM*, *COBL*, *COL4A2*, *COL22A1*, *FMN2*, *KIAA1211*, *PDE4DIP*, *ROBO1*, and *SLIT2*. These proteins are known for their functions related to cytoskeleton formation and rearrangements associated with cell–cell and cell–matrix interactions [5]. These genetic alterations may play a role in the metastatic process of SCLC, though the exact mechanisms and their implications are still being studied. A recent paper demonstrated that *CCN1/2* (Cellular communication network factor 1/2) is regulated by transcription factor YAP1 to inhibit SCLC metastasis. CCN1/2 blocked the actin polymerization and thereby inhibited the migration of SCLC cells [56]. Another group identified CUL5 (Cullin5) and SOCS3 (suppressor of cytokine signaling 3) from the pooled CRISPR/Cas9 library as candidate regulators of SCLC metastasis. The depletion of CUL5/SOCS3 stabilized integrin ß1 and promoted metastasis through focal adhesion kinase/SRC signaling pathway, which predicts the potential benefit for CUL5-deficient SCLC patients from receiving SRC inhibitor treatment [57].

## 3. SCLC Heterogeneity and Phenotypic Switching

**Figure 1:**
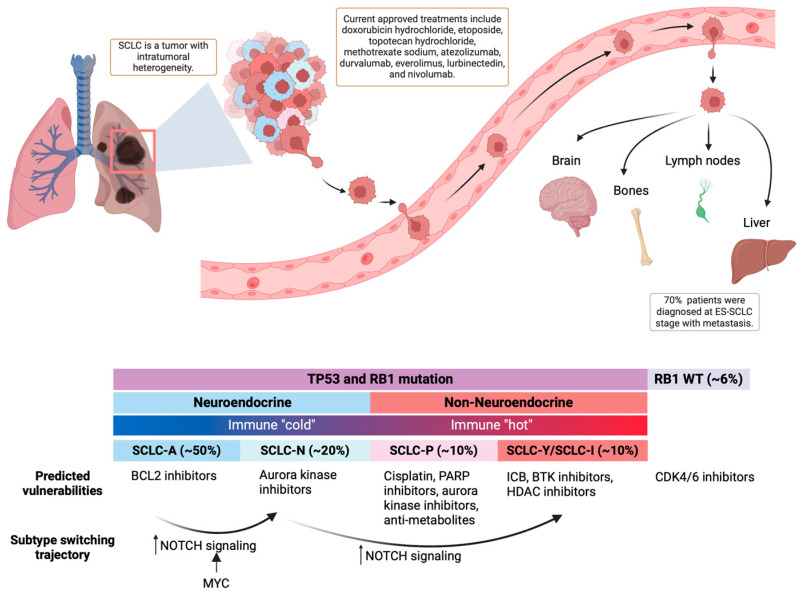
Basics of SCLC. **Top:** SCLC is a malignancy with high plasticity and early metastasis. **Bottom:** SCLC can be classified into four major subtypes that differ in neuroendocrine differentiation, immune response, and treatment vulnerabilities.

In recent years, research has unveiled that SCLC should no longer be analyzed and treated as a single disease. It is a malignancy with unique subtypes, each with distinct genomic profiles, including immune-related genes, and therapy responses. Over 30 years ago, human SCLCs were initially divided into two subgroups: the neuroendocrine (NE) subgroup, originating from pulmonary neuroendocrine cells, and the non-neuroendocrine (variant) (non-NE) subgroup [58]. Subsequent studies have indicated the presence of non-NE tumor cells within single SCLC cell lines and mouse models, revealing intratumoral heterogeneity [41,59]. The plasticity of tumor cells and their ability to transform between subtypes poses a significant challenge in targeting SCLC and may contribute to poor treatment outcomes and relapse (Figure 1).

### 3.1. Intertumoral Subtypes

Over the past decade, increased clinical cases unveiled a more complex landscape of SCLC subtypes. The computational modeling of transcriptomics data has defined SCLC subtypes using the expression of four key transcription factors: achaete-scute homolog 1 (ASCL1), Neuronal Differentiation 1 (NEUROD1), POU class 2 homeobox 3 (POU2F3), and Yes-Associated Protein 1 (YAP1) [60]. So far, no reported SCLC case lacks the expression of any of these four genes.

- Neuroendocrine SCLC has a high expression of NE markers, including Synaptophysin (SYP), Chromogranin-A (CHGA), and Neural cell adhesion molecule 1 (NCAM1 or CD56). NE SCLC can be divided into two subtypes:
  ο SCLC-A: This neuroendocrine subtype is characterized by high ASCL1 expression and accounts for approximately 50% of primary SCLC cases [60]. ASCL1 is an NE-lineage-specific transcription factor essential for SCLC tumorigenesis [61]. It exhibits super-enhancers associated with genes like MYCL1, NFIB, BCL2, NKX2-1, FOXA1, and FOXA2 [61]. Gene ontology analysis reveals enrichment in neuronal systems, potassium channel genes, and epithelial cell differentiation [61,62].
  ο SCLC-N: This neuroendocrine subtype, comprising 20% of primary SCLC cases, is characterized by high NEUROD1 expression and low ASCL1. SCLC-N often exhibits super-enhancers associated with NEUROD1 and the oncogene MYC [61]. This subtype may respond to chemotherapy but can develop resistance [31].
- Non-NE SCLC has low expression of both ASCL1 and NEUROD1 and can be divided into two subtypes:
  ο SCLC-P: Characterized by high POU2F3 expression, it exhibits the unique expression of other transcription factors, including SOX9 and ASCL2, and the tyrosine kinase receptor insulin-like growth factor 1 receptor (IGF1R) [63].
  ο The remaining SCLC tumors have low expressions of ASCL1, NEUROD1, and POU2F3. Two putative subtypes are SCLC-Y and SCLC-I.
    - SCLC-Y: With high YAP1 expression, it is sensitive to CDK4/6 inhibitors [64].
    - SCLC-Inflamed: This subtype is characterized by an inflamed gene signature (including immune checkpoints and HLAs), making it benefit from immunotherapy, but this subtype is not uniquely defined by YAP1 expression [6,65].

Multiple studies showed that patients with different SCLC subtypes have different prognoses. Based on surgery-resected patient tumor samples, better prognoses after the primary tumor surgery were observed in the low-NE group compared to the high-NE group [66,67]. More specifically, patients with the SCLC-Y subtype had the best prognosis, while patients with the SCLC-A subtype had the worst [68]. 

### 3.2. Intratumoral Heterogeneity and Evolution

The study of intratumoral heterogeneity and the evolution of SCLC has revealed a complex and dynamic landscape. In recent years, it has been observed that the majority of human and mouse SCLC tumors consist of multiple subtypes, indicating a high degree of tumor plasticity [59,62]. Tumor subpopulations within the same patients have shown dynamic changes both before and after treatment, highlighting the adaptability of SCLC. 

Research by Borromeo and others demonstrated that *Ascl1*, but not *Neurod1*, is necessary for tumor formation in mouse models with *Tp53/Rb1/Rbl2^lox/lox^* mutations [61]. This suggests that ASCL1 expression might serve as a precursor to both SCLC-A and SCLC-N subtypes, with SCLC-N potentially evolving from an ASCL1-expressing state.

Further investigations have shown that MYC plays a crucial role in driving the evolution of subtypes from SCLC-A to SCLC-N and SCLC-Y [39]. MYC activates the Notch pathway, promoting the emergence of SCLC-N and SCLC-Y subtypes from a cell of origin that initially expresses ASCL1. Simultaneously, it propels the SCLC-P subtype from a cell of origin that is not a pulmonary neuroendocrine cell (PNEC), club cell, or AT2 cell [39].

Studies conducted on early-stage tumor cells from mouse models (*Tp53/Rb1^lox/lox^* or RPM mice) cultured over time have revealed a temporal subtype transition from SCLC-A-dominant to SCLC-Y-dominant. Initially, cells expressed high levels of ASCL1 and other NE markers within the first 4–7 days of culture. However, by days 11–21, they exhibited high levels of non-NE markers. This MYC-driven subtype evolution is dependent on Notch pathway activation [39].

Considering that Notch pathway loss-of-function mutations have been reported in approximately 25% of human SCLCs [12], it has been proposed that SCLC tumor cells with defective Notch signaling remain in an NE-high state, while tumor cells with intact and activated Notch are reprogrammed by MYC to a non-NE state.

Although the above studies all showed an NE to non-NE transition, so far, there is no reported non-NE to NE transition. Advanced lineage-tracing techniques during SCLC development are expected to provide a better understanding of the SCLC evolution and help in the development of subtype-targeted or plasticity-targeted therapeutic strategies.

### 3.3. The Immune Microenvironment in SCLC Subtypes

Immunotherapies for SCLC primarily involve immune checkpoint blockers (ICBs) targeting PD1, PD-L1, and CTLA4. However, the clinical benefits of immunotherapy in SCLC have been limited, with fewer than 20% of patients experiencing substantial improvements [69]. This can be attributed to the overall “cold” status of the immune environment in neuroendocrine SCLC. SCLC typically exhibits a low expression of class 1 major histocompatibility complex (MHC) antigens and a limited infiltration of cytotoxic immune cells compared to other tumor types [70].

As researchers have characterized SCLC subtypes, questions have arisen regarding whether the immune microenvironment varies among these subtypes and whether they respond differently to ICB. There is substantial evidence indicating increased MHC I expression and immunogenicity in non-NE subtypes [6,65,71]. SCLC subtypes display distinct immune properties. SCLC-N, for instance, exhibits the lowest expression of immune-related genes, including those involved in MHC and antigen presentation, immune checkpoints, and natural killer (NK) cells. In contrast, the SCLC-P subtype has the highest expression of immune-related genes [71]. Studies have shown that low MHC I antigen presentation in NE SCLC is associated with the epigenetic silencing of TAP1 by EZH2. The inhibition of EZH2 can reverse this process, converting NE SCLC into an antigenic non-NE phenotype [65].

As previously mentioned, SCLC-Inflamed (SCLC-I) has shown elevated immune infiltrate, including T cells, NK cells, and macrophages, as well as a high cytolytic activity score [6]. SCLC-I does not exhibit prevailing signatures of ASCL1, NEUROD1, POU2F3, and, in some cases, YAP1. Interestingly, drug response analysis has shown that SCLC-I is more resistant to cisplatin treatment. Unsurprisingly, SCLC-I has experienced greater benefits from the combination of ICBs, such as atezolizumab (an anti-PD-L1 antibody), with chemotherapy [6]. These findings highlight the importance of understanding the immune microenvironment and its variations among SCLC subtypes when developing effective therapeutic strategies.

### 3.4. Therapeutic Vulnerabilities in SCLC Subtypes

The distinct profiles of genomic alteration, epigenetic regulation, and the immune microenvironment observed across SCLC subtypes have raised the possibility of developing unique subtype-specific therapeutic strategies. In vitro drug response data from SCLC cell lines have provided valuable insights into potential vulnerabilities and treatment options for each subtype:

SCLC-A (ASCL1-high): This subtype appears to have a high expression of BCL2 protein and is predicted to be sensitive to BCL2 inhibitors [6]. Targeting the BCL2 protein, which plays a role in inhibiting cell death, may be a viable therapeutic approach for SCLC-A.

SCLC-N (NEUROD1-high): SCLC-N has shown increased sensitivity to aurora kinase inhibitors, but also resistance to standard cisplatin treatment [6]. Aurora kinase inhibitors can disrupt cell division and may offer a promising treatment strategy for SCLC-N.

SCLC-P (POU2F3-high): SCLC-P is most sensitive to cisplatin treatment and shows sensitivity to poly-ADP ribose polymerase (PARP) inhibitors and anti-metabolites, such as anti-folates, as well as aurora kinase inhibitors [6,31,62]. PARP inhibitors target DNA repair processes, while anti-metabolites interfere with the production of DNA and RNA in cancer cells.

SCLC-I (SCLC-Inflamed): This subtype shows resistance to cisplatin but is predicted to benefit from ICBs and Bruton’s tyrosine kinase (BTK) inhibitors [6]. Patients with MHC I^hi^ showed significantly more durable responses to ICBs and increased overall survival [65]. Also, due to the most mesenchymal phenotype among all subtypes, it is predicted that this subtype will also benefit from treatment strategies targeting EMT, such as HDAC inhibitors [6].

Beyond the drug response analysis, the distinct expression patterns of surface proteins in SCLC subtypes present an alternative avenue for targeted therapies by antibody—drug-conjugates (ADCs) and chimeric antigen receptor (CAR) strategies. By tailoring treatments to the unique characteristics of each SCLC subtype, we can improve therapeutic outcomes and address the challenges posed by SCLC’s heterogeneity and phenotypic switching.

## 4. Current Treatment

Therapeutic options for SCLC have seen little progress over the last three decades, with the major strategies centered around conventional chemotherapy and radiation therapy. The standard of care for SCLC continues to involve platinum-based alkylating agents, such as cisplatin or carboplatin, typically in combination with topoisomerase inhibitors like etoposide or irinotecan.

SCLC is classified into two stages: limited-stage (LS, stage I-III) and extensive-stage (ES, stage IV). Although surgery is not considered the main treatment option for SCLC due to the early metastasis, for patients with LS-SCLC at clinical stage I-IIA (about 5% in patients with SCLC), surgical resection is the recommended primary treatment according to the NCCN Guidelines for SCLC. For those with LS-SCLC stage IIB-IIIC, the standard practice recommends concurrent or sequential radiotherapy directed at the thorax and mediastinum, alongside platinum-based chemotherapy [2]. For extensive-stage SCLC (ES-SCLC), the initial course of treatment predominantly revolves around chemotherapy. The role and merit of thoracic radiation and prophylactic cranial irradiation (PCI) in ES-SCLC therapy remain subjects of contention and are not universally advised for all patients [72].

A significant hurdle in advancing therapeutic options for SCLC was the perception of this cancer as a “homogenous” tumor, resulting in a standard approach involving a combination of chemotherapy and radiotherapy for all SCLC patients [42]. This categorization, which has persisted for years due to the intertumoral pathological similarities, has contributed to the slow progress. Additionally, the list of drugs approved for the specific treatment of SCLC remains exceptionally short as of April 2024. Apart from common chemotherapy drugs like Doxorubicin Hydrochloride, Etoposide Phosphate, Topotecan Hydrochloride, and Methotrexate Sodium, only five drugs are approved for SCLC treatment.

Everolimus (Afinitor, Novartis) is an mTOR inhibitor approved for the treatment of adult patients with progressive, well-differentiated non-functional, neuroendocrine tumors (NETs) of gastrointestinal (GI) or lung origin with unresectable, locally advanced or metastatic disease [73]. The inhibition of mTOR blocks the translation of genes that promote tumor cell growth and survival, including angiogenesis and metabolism.

Lurbinectedin (ZEPZELCA, Pharma Mar S.A.) is a DNA alkylating agent that causes more double-strand DNA breaks and cell death in hyper-proliferating tumor cells. It has been recently approved for adult patients with metastatic SCLC upon or after platinum-based treatment. The complete mechanism of action remains elusive, but it was revealed that Lurbinectedin can also inhibit hyperactivated RNA polymerase II, resulting in reduced oncogene expression [74,75]. Lurbinectedin was granted accelerated approval after the multicenter PM1183-B-005-14 trial (Study B-005; ClinicalTrials.gov identifier NCT02454972) showed a 35% overall response rate was achieved among all patients in the trial, with a 5.3-month median response duration [74]. Despite its accelerated approval as a new monotherapy for metastatic SCLC, the combination of lurbinectedin with doxorubicin did not achieve an improved overall survival compared with the current second-line standard treatment of topotecan or cyclophosphamide, doxorubicin, and vincristine (CAV) in the multicenter, randomized, controlled, phase 3 ATLANTIS study [76]. These trials indicate that lurbinectedin as an active agent in SCLC could be further developed in both monotherapy and combinational therapy against different stages of SCLC.

Immune checkpoints regulate the immune system, and they fall into two major groups: stimulatory and inhibitory checkpoints. Inhibitory checkpoints maintain self-tolerance, making sure healthy cells are not destroyed by the activated T cells. However, cancer cells also exploit those inhibitory immune checkpoints to evade the immune response. Two inhibitory immune checkpoint receptors have been actively studied in the past few years: cytotoxic T-lymphocyte-associated antigen 4 (CTLA4; or CD152) and programmed cell death protein 1 (PD1; or CD279). Immune checkpoint inhibitors such as ipilimumab, nivolumab, pembrolizumab, durvalumab, tremelimumab, and ulocuplumab are at the forefront of immunotherapy and have achieved approvals for certain cancer types, varying from SCLC to hematologic malignancies [77].

Atezolizumab (TECENTRIQ) is an anti-PD-L1 monoclonal antibody approved with carboplatin and etoposide as a first-line treatment for ES-SCLC. By binding to programmed death-ligand 1 (PD-L1) on some tumor cells, atezolizumab inhibits the interaction between programmed death receptor 1 (PD1), inhibitory receptor on the surface of activated T cells, and PD-L1 and further prevents the immune evasion of tumor cells. Atezolizumab and carboplatin combination for ES-SCLC was approved after the IMPower133 (NCT02763579) trial, which showed significant improvement in the overall survival and progression-free rate (PFS) compared with the placebo group [69,78].

Like atezolizumab, durvalumab (IMFINZI) is another PD1/PD-L1 checkpoint monoclonal antibody inhibitor that binds PD-L1. It was approved with etoposide and either carboplatin or cisplatin as a first-line treatment for ES-SCLC based on the result in CASPIAN, a randomized, multicenter, active-controlled, open-label trial (NCT03043872). The combination treatment showed efficacious clinical outcomes in overall survival but not antibody-dependent cytotoxicity among patients. Both atezolizumab (single-mutation) and durvalumab (three mutations) are IgG1 isotypes and have engineered Fc domains to bypass the attack of PD-L1-expressed T cells and other antibody-dependent cytotoxicity [79]. The undesirable antidrug antibody (ADA) formation indicates the drug’s immunogenicity and it can affect the drug’s pharmacokinetics, pharmacodynamics, and efficacy [79]. The complete mechanisms of immunotherapy that elicited ADA formation remain elusive, but due to the different designs of atezolizumab and durvalumab, they have different ADA formation incidents. Atezolizumab has a reported treatment-emergent antidrug antibody (ADA) of 30% to 48%, while durvalumab’s is only 3.1% [80].

Besides targeting PD-L1, another strategy is to target PD1 receptors on the immune cells to block PD1/PD-L1 interaction. Nivolumab (Opdivo) is an anti-PD1 monoclonal antibody approved in the U.S. as a third-line treatment for metastatic SCLC with progression upon or after platinum-based chemotherapy and at least one other line of therapy. The accelerated approval was based on the efficacy outcome measures from the metastatic SCLC patients in CheckMate-032 (NCT01928394), a multicenter, open-label trial in patients with metastatic solid tumors. The overall response rate (ORR) was 12% (95% CI: 6.5, 19.5). Responses were durable for 6 months or longer in 77%, 12 months or longer in 62%, and 18 months or longer in 39% of the 13 responding patients [81].

## 5. Promising Treatment Options for SCLC

Besides the approved drugs for SCLC treatment, researchers are coming up with creative ways to target SCLC, which can be divided into three major areas: (1) targeted therapy (including drug conjugates and small molecule inhibitors), (2) immunotherapy, and (3) chemotherapy. Other innovative strategies include systemic gene therapy utilizing lipid nanoparticles that encapsulate plasmid to express tumor suppressor gene TUSC2 [82].

### 5.1. Targeted Therapies

Antibody–drug conjugates (ADCs) are innovative biopharmaceutical drugs that combine the advantages of immuno- and chemotherapy [Table 1]. Highly specific monoclonal antibodies against antigens presented on tumor cells are chemically linked to active antitumor agents, which significantly decreases systematic toxicity. The high selectivity and high lethality against tumor cells, while sparing healthy cells, have made ADC drugs a very powerful and promising cancer treatment option. So far, there are several ADC drugs on the market and about 100 ADCs in clinical trials for various cancer types. For SCLC, although there is no approved ADC, there are several in clinical trials.

One promising antigen is B7-H3 (CD276), a transmembrane immune checkpoint protein selectively overexpressed in cancer cells to promote immune evasion. High B7-H3 expression has been detected in 65% of SCLC patients, making it a candidate target for immunotherapy and targeted therapy [83]. Currently, there are three B7-H3-directed ADCs in SCLC clinical trials. Ifinatamab Deruxtecan (I-DXd) is in phase III trial for patients with relapsed SCLC based on its encouraging ORR of 52.4% from phase I/II subgroup analysis [84,85]. The other two B7-H3-directed ADCs are HS-20093 and ABBV-155 (Mirzotamab clezutoclax; Mirzo-C), which have a BCL-X_L_ inhibitor payload [86].

Other antigens targeted in the ADCs include SEZ6, a cell-surface protein that is highly expressed in neuroendocrine tumors including SCLC [87] and TROP2, a glycoprotein overexpressed in epithelial tumors like SCLC [88]. DLL3, a ligand that inhibits Notch pathway activation and is selectively overexpressed in SCLC (~80%), was proposed to be a promising ADC target based on the efficacy results of the Rova-T phase I trial [89]. Unfortunately, the following trials of Rova-T failed to demonstrate efficacy, and the development of the drug was discontinued [43], which leads to the question of whether DLL3 is a valid target. Currently, there are still multiple anti-DLL3 agents in clinical trials, including ZL-1310 (anti-DLL3 ADC), BI 764532 (bispecific DLL3/CD3 T cell engager), HPN328 (anti-DLL3 T cell engager), and DLL3-directed CAR-T and CAR-NK therapies. The results of these trials will provide a clear understanding of DLL3 expression as a biomarker for SCLC treatment.

A new format of “drug conjugates” has started to exploit interactions other than antigen–antibody recognition to deliver the payload. CBX-12 is an alphalex peptide drug conjugate (PDC) that consists of a pH-sensitive alphalex peptide, a linker, and a topoisomerase inhibitor exatecan. In the tumor microenvironment, where the pH is lower than 7.0, the peptide forms an alpha helix that inserts into the cell membrane to release the linker and payload [90].

Another major advance in the targeted therapy of SCLC is the development of new small molecule inhibitors and agonists, which target various tumor activities including histone modification (HDAC, EZH1/2), cell cycle regulation (CDK2/4/6), DNA damage repair (ATR, PARP), angiogenesis (VEGFR, PDGFR), proteasome activity, and other important kinase activities (e.g., Aurora, PERK, and PP2A) [Table 1].

### 5.2. Immunotherapies

**Figure 2:**
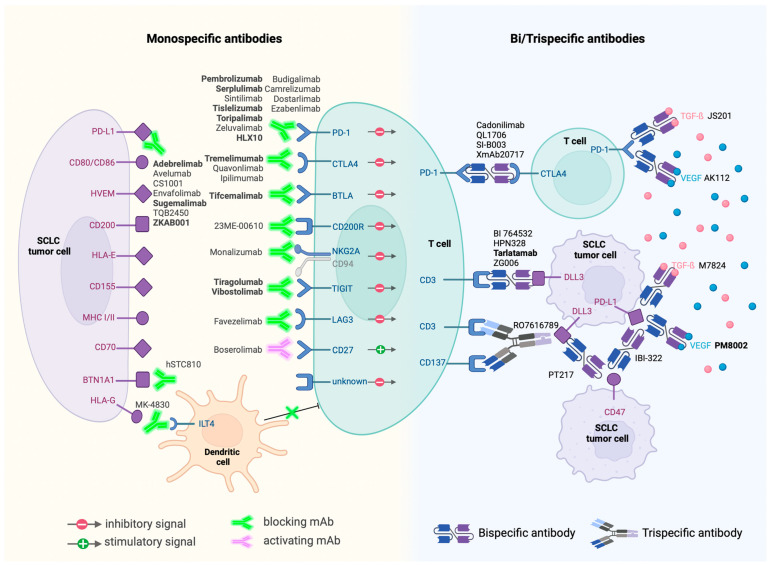
Immunotherapies currently in clinical trials and their targets. Antibodies in bold are currently in phase III trials.

Despite SCLC’s reputation of being an “immune desert”, significant progress has been made with FDA-approved monoclonal antibodies targeting PD1 and PD-L1. These agents, often used in combination with chemotherapy, have shown positive outcomes, sparking further interest and development in this area (Figure 2).

Monoclonal Antibodies: A significant aspect of current research focuses on monoclonal antibodies that modulate the immune checkpoints or influence the immune response directly. As of April 2024, around fifty monoclonal antibodies are under clinical evaluation. The exploration extends beyond PD1 and PD-L1 targets to include cytotoxic T-lymphocyte antigen 4 (CTLA-4), butyrophilin 1A1 (BTN1A1), T cell immunoreceptor with Ig and ITIM domains (TIGIT), T cell immunoglobulin domain and mucin domain 3 (TIM3), CD200 receptor 1 (CD200R1), B- and T-lymphocyte attenuator (BTLA), CD94/NK group 2 member A (NKG2A), immunoglobulin-like transcript 4 (ILT4) receptor, CD27, and lymphocyte-activation gene 3 (LAG3). This broadening of targets underscores the evolving understanding of the immune landscape in SCLC.

Next-Generation Antibodies: The advent of bispecific monoclonal antibodies marks a notable advancement. These antibodies, capable of targeting two antigens simultaneously, exhibit increased target-binding efficiency and potential for enhanced antitumor activity. Examples include the bispecific checkpoint inhibitors PSB 205 (targeting PD1 and CTLA-4) and XmAb22841 (targeting CTLA-4 and LAG3).

BiTEs and Beyond: Among bispecific antibodies, BiTEs (Bispecific T cell Engagers) like AMG-757, targeting the inhibitory Notch pathway ligand DLL3, have shown promise. Early studies indicate that AMG-757 can effectively redirect T cells to eliminate DLL3-positive cancer cells [44,91].

Moreover, bispecific antibodies are exploring combinations beyond ICB, venturing into areas such as anti-angiogenesis. One example is PM8002, a bispecific antibody that combines PD-L1 inhibition with VEGF blockade. The early phase II results of PM8002 and paclitaxel combination treatment showed an impressive overall response rate of 72.7% in immunotherapy-naïve patients [92].

Other innovative immunotherapy agents include cytokines and agonists to stimulate immune response (IL7, IL12, IL15, and CD137 agonist), CAR-T (DLL3-directed and GD2-directed), CAR-NK (DLL3-directed), and small molecule inhibitor and antisense oligonucleotides (ASOs) to relieve immunosuppression. RRx-001 is a small molecule Myc inhibitor currently in phase III trial. The proposed mechanism of action is that RRx-001 downregulates the expression of immune checkpoints CD47 and PD-L1 to sensitize macrophages and T cells through c-Myc inhibition [93]. One clinical ASO candidate is AZD8701, which degrades Forkhead-box P3 transcription factor (FOXP3) mRNA that promotes the regulatory T cell’s immunosuppression activity [94]. This first-in-class strategy to regulate immune response through ASO provides a novel approach to fight against SCLC.

## 6. Advance in Early Lung Cancer Diagnosis and Assessment of Therapy Response

Despite the advancement in understanding the biological pathways and identifying therapeutic targets, there is still a lack of non-invasive and sensitive diagnosis and screening tools for lung cancer. One contributing factor to poor prognoses in SCLC is the late diagnosis, making the conventional treatments less effective. Another factor is that the assessment of therapy is often limited by invasive biopsy procedures and complex tumor dynamics. Tumors can have delayed shrinkage and transiently enlarge due to inflammation, especially after immunotherapies, which makes it hard to interpret the treatment response simply from the serial imaging [95]. In the past decade, the technological advances in measuring and analyzing circulating tumor DNA (ctDNA) or other markers (exosomes) in blood have shown the potential of “liquid biopsy” for early detection and therapy assessment in both leukemia and solid malignancies, including lung cancer [96,97]. Compared with traditional tissue biopsy, liquid biopsy is less limited by tumor accessibility, sampling frequency, and complicated tumor dynamics.

Although there is currently no ctDNA-based diagnostic tool for SCLC on the market, the FDA approved Guardant360 CDx as a companion diagnostic for NSCLC patients with epidermal growth factor receptor (EGFR) alterations who may benefit from treatment with osimertinib (Tagrisso) in August 2020 [98]. The approval of Guardant360 CDx paved the way for repurposing the biomarkers into diagnostic and prognostic tools in all solid neoplasms, especially in lung tumors.

Besides genomic analysis such as Guardant360 CDx, epigenetic-based ctDNA testing is potentially also a powerful screening tool. The hypermethylation of Septin9 in ctDNA is observed in various cancers, including colorectal cancer and lung cancer [99]. mSEPT9 test was approved by the FDA as a commercial test for colorectal cancer (CRC)-screening tests after clinical trials. LUNAR-2, which combines the genomic and epigenomic ctDNA analyses, is currently under clinical trials to study the risk stratification in lung cancer screening [100]. SUMMIT, another early detection blood test clinical trial, is ongoing in the UK to validate the blood test in individuals with high-risk lung cancer [101].

Still, despite the advantages of the liquid biopsy, the development of biological techniques, and the promising results in clinical trials, there is uncertainty about the ctDNA-based blood test for lung cancer, including its accuracy and effectiveness [102]. With careful biomarker design, rational clinical implementation, and result interpretation considering unique lung tumor characteristics, ctDNA-based assays are likely to have an impact on lung cancer care.

## 7. Remaining Challenges and Future Directions

Although most patients respond very well to primary treatments like chemotherapy and radiation, relapse and developed drug resistance are still major clinical challenges. The heterogenous nature of SCLC contributes to this phenomenon as different subtypes display distinct resistance to treatment. The less-sensitive cells that survive from initial treatment are the origins of the relapsed tumor. To date, many efforts have focused on exploring drug resistance in SCLC. Besides the previously mentioned EZH2-SLFN11 axis [46], another driver of chemoresistance identified is MYCN and the potential of inhibiting USP7 to restore chemosensitivity [103]. Despite these preliminary results, drug resistance in SCLC remains a big obstacle. Combinational regimens might be one strategy to increase efficacy while reducing the likelihood of developing resistance. Currently, there are many combinational treatments and a few targeted therapies for refractory or relapsed SCLC in clinical trials [Table 1], including one EZH2 inhibitor, PF-06821497. The results of these trials will provide valuable insights into developing strategies for fighting chemoresistance.

Treatment toxicity should also be carefully evaluated and monitored in clinical practice and in trials. One example is the failure of Rova-T with the toxic payload PBD. Unlike other ADCs utilizing topoisomerase inhibitor or tubulin inhibitor monomethyl auristatin E (MMAE), PBD is more potent yet toxic. The linker of Rova-T is also predicted to have early cleavage, resulting in PBD systematic exposure [104].

- It is essential to recognize that SCLC management is evolving, with ongoing research and clinical trials exploring novel and potentially more effective therapies for this aggressive cancer. Recent insights into the heterogeneous nature of SCLC, as well as advancements in understanding its plasticity, offer the potential for tailored and targeted treatment approaches. These may encompass subtype-specific therapies, immunotherapies, and innovative treatments based on epigenetics and other cutting-edge approaches. Here, we propose a few future directions for studying and targeting SCLC:
- It is exciting to observe the declining incidence rate of SCLC with the help of global tobacco control programs. It is crucial to continue public education emphasizing smoking as the primary cause of SCLC and advocating for reduced tobacco consumption.
- The identification of predictive biomarkers will be crucial for treating SCLC. Although different subtype’s therapeutic vulnerabilities have been predicted with drug library screening [62], the exact difference among subtypes should be more closely investigated. The inclusion of subtype-specific markers (ASCL1, NEUROD1, POU2F3, and maybe YAP1) for immunohistochemistry staining besides neuroendocrine markers, such as SYP and NCAM1, will benefit the physicians in diagnosing patients with specific SCLC subtypes and predicting the potential treatment response. Stratifying patients based on molecular subtypes should also be incorporated into clinical trial design. The failure of certain targets in the clinical trials might be due to not targeting the proper patient subpopulation. Tumor shapeshifting after treatment, especially chemotherapy, should also be considered when designing clinical trials. One example is SLFN11, which is utilized as a predictive marker for PARP1/2-targeted therapies [Table 1].
- Another big direction will be to improve the immunotherapy response. Since non-neuroendocrine subtypes (especially triple-negative for ASCL1, NEUROD1, and POU2F3) showed more immune infiltration, identifying the genes switching neuroendocrine SCLC to non-neuroendocrine SCLC will be critically important to achieve durable immune therapy response by directing immune “cold” NE to immune “hot” non-NE SCLC. One major player for the switch is the activation of the MYC-Notch signaling pathway, which has been shown to drive the SCLC-A subtype to SCLC-N and eventually to SCLC-Y [39]. Treatments targeting this pathway and other mechanisms underlying the NE-to-non-NE switch should be investigated.
- Considering the general immune “cold” phenotype in the classic neuroendocrine SCLC, immunotherapy, especially monospecific immune checkpoint inhibitors alone, might not be the best strategy, as shown by the moderate clinical ORRs, but targeting overexpressed antigens with proper payload and antigen-directed T cell engagers might have better efficacy. Another approach is to explore the combinational treatment of ICBs with non-NE-induction treatment.
- The approval of atezolizumab and durvalumab as a first-line treatment with platinum-based chemotherapy ignites the exploration of combined regimens. It provides the opportunities to target tumors while potentially bypassing the resistance; however, it also brings challenges: finding the best combination in this heterogenous and shapeshifting malignancy and determining the best dosage schedule when designing clinical trials.
- Due to the plasticity and heterogeneity of SCLC, models like patient-derived xenograft will be a powerful tool to monitor the subtype transition before, during, and after the treatment, to develop a more personalized treatment plan. Also, validating the preliminary results obtained from murine models and human SCLC cell lines in these patient-derived xenograft models will increase the probability of successful laboratory-to-clinic translation.

## Figures and Tables

**Table 1:** Targeted therapies for SCLC currently in clinical trials.

Therapeutic Class		Target(s)	Drug	Trial ID	Phase	Disease Conditions
ADC and ADC like		B7-H3	ABBV-155 (Mirzo-C)	NCT03595059	1	Relapsed/refractory solid tumors
			Ifinatamab Deruxtecan	NCT05280470	2	ES-SCLC
				NCT06203210	3	Relapsed/refractory SCLC
			HS-20093	NCT06052423	2	ES-SCLC
		SEZ6	ABBV-011	NCT03639194	1	Relapsed/refractory SCLC
			ABBV-706	NCT05599984	1	Advanced solid tumors with SEZ6 expression
		Carbonic anhydrase IX (CAIX)	89Zr-DFO-girentuximab	NCT05563272	2	CAIX-positive solid tumors
		EGFRxHER3 bispecific antibody	BL-B01D1	NCT05924841	2	ES-SCLC
		pH-sensitive peptide	CBX-12	NCT04902872	1,2	Advanced or metastatic refractory solid tumors
		GD2	GD2-SADA:177Lu-DOTA Complex	NCT05130255	1	GD2-expressing solid tumors
		Somatostatin receptor	177-Lu Dotatate	NCT05142696	1	Newly diagnosed ES-SCLC
		heat shock protein 90 (HSP90)	PEN-866 Sodium	NCT03221400	1,2	Advanced solid tumors
		Trop-2	Sacituzumab govitecan	NCT04826341	1,2	SCLC
			SKB264	NCT04152499	1,2	Refractory advanced solid tumors
		DLL3	ZL-1310	NCT06179069	1	SCLC
Small molecule inhibitors	Kinase inhibitor	CDK2 inhibitor	PF-07104091	NCT04553133	1,2	SCLC
		Dual CDK4/CDK6i	Abemaciclib	NCT04010357	2	Chemo-refractory, RB1 wild-type ES-SCLC
		Aurora A inhibitor	Alisertib	NCT06095505	2	ES-SCLC
			JAB-2485	NCT05490472	1,2	Advanced solid tumors
		Aurora B inhibitor	AZD2811	NCT04745689	2	SCLC
		VEGFR2 inhibitor	Apatinib	NCT04683198	2	ES-SCLC
		ATR inhibitor	Berzosertib	NCT04826341	1,2	SCLC
				NCT02595931	1	Metastatic or unresectable solid tumors
				NCT03896503	2	SCLC
				NCT02487095	1,2	SCLC
			Bevacizumab	NCT05588388	2	ES-SCLC and liver metastases
				NCT04730999	2	ES-SCLC
				NCT02734004	1,2	Advanced solid tumors
			Ceralasertib	NCT04699838	2	ES-SCLC
			Elimusertib	NCT04491942	1	Advanced solid tumors
				NCT04514497	1	Advanced solid tumors
			SC0245	NCT05731518	1,2	ES-SCLC
		WEE1 inhibitor	Debio 0123	NCT05815160	1	Relapsed/refractory SCLC
		PERK inhibitor	HC-5404-FU	NCT04834778	1	Advanced solid tumors
		EGFR inhibitor	HLX07	NCT05354700	2	ES-SCLC
		FAK inhibitor	IN10018	NCT06030258	1,2	ES-SCLC
		PLK inhibitor	Onvansertib	NCT05450965	2	Relapsed/refractory SCLC
		Pan-VEGFR inhibitor	Cediranib Maleate	NCT02498613	2	Advanced solid tumors
			lenvatinib	NCT04938817	1,2	ES-SCLC
				NCT04924101	2	ES-SCLC
				NCT05384015	2	ES-SCLC
		TAM receptors and VEGFR2 inhibitor	Sitravatinib	NCT05228496	2	ES-SCLC
		Pan-VEGFR and PDGFR inhibitor	Vorolanib	NCT03583086	1,2	Refractory thoracic tumors
				NCT04373369	2	ES-SCLC
		Multiple kinase inhibitors (VEGFR, PDGFR, c-Kit, Aurora B, and CSF-1R)	Chiauranib	NCT05271292	1,2	Relapsed/refractory SCLC
				NCT05371899	NA *	SCLC
				NCT04830813	3	SCLC
		Multiple kinase inhibitors (VEGFR, FGFR1, and CSF-1R)	Surufatinib	NCT04579679	2	NET
				NCT04579757	1,2	Advanced solid tumors
				NCT05668767	2	ES-SCLC
				NCT04996771	1,2	SCLC
				NCT05882630	1,2	ES-SCLC
				NCT05509699	2	ES-SCLC
				NCT05595889	2	SCLC
				NCT05527821	2	Advanced solid tumors
		multi-kinase inhibitor (Aurora A/B, JAK, FGFRs and VEGFRs)	TT-00420	NCT04742959	1,2	Advanced solid tumors
				NCT05253053	1,2	Advanced solid tumors
		Multiple kinase inhibitors (VEGFR, FGFR, PDGFR, c-Kit, and Ret)	AL3818 (Anlotinib)	NCT04165330	1,2	Advanced solid tumors
				NCT04985851	NA *	ES-SCLC
				NCT05942508	1b	LS-SCLC
				NCT05896059	2	ES-SCLC
				NCT04757779	2	Relapsed/refractory SCLC
		Multiple kinase inhibitors (Aurora B, FGFR, and VEGFR)	AL8326	NCT05363280	2	SCLC
	Epigenetic regulator inhibitor	LSD1 inhibitor	Bomedemstat	NCT05191797	1,2	SCLC
			CC-90011	NCT03850067	1	ES-SCLC
			Iadademstat (ORY-1001)	NCT05420636	2	Relapsed/refractory SCLC
		EZH2 inhibitor	PF-06821497	NCT03460977	1	Relapsed/refractory SCLC
			XNW5004	NCT06022757	1,2	Advanced solid tumors
	Other inhibitors	PARP1/2 inhibitor	Fluzoparib (SHR-3162)	NCT04400188	1,2	Relapsed/refractory SCLC
			HTMC0435	NCT05728619	1,2	Recurrent ES-SCLC
			IMP4297(senaparib)	NCT04434482	1,2	Advanced solid tumors
			Niraparib	NCT05718323	2	SLFN11-positive, ES-SCLC
				NCT03830918	1,2	ES-SCLC
				NCT04701307	2	SCLC
				NCT03221400	1,2	Advanced solid tumors
			Olaparib	NCT04538378	2	EGFR-mutated adenocarcinomas that transform into SCLC or NE tumors
				NCT02734004	1,2	Advanced solid tumors
				NCT04624204	3	Treatment-naïve LS-SCLC
				NCT04728230	1,2	ES-SCLC
				NCT03923270	1	SCLC
				NCT02769962	1,2	Relapsed/refractory SCLC
				NCT02498613	2	Advanced solid tumors
			Pamiparib (BGB-290)	NCT05483543	2	LS-SCLC
			RP12146	NCT05002868	1	Locally advanced or metastatic solid tumors
			Rucaparib	NCT04209595	1,2	Solid tumors and small cell cancers
				NCT03958045	2	SCLC
			Talazoparib	NCT04334941	2	SLFN11 Positive SCLC
				NCT03672773	2	ES-SCLC
		PP2A inhibitor	LB-100	NCT04560972	1	ES-SCLC
		Exportin-1 (nuclear export) inhibitor	Selinexor	NCT05975944	1,2	ES-SCLC

NA * (not applicable) is used to describe trials without FDA-defined phases.

## Data Availability

Not applicable.
